# Tooth-Derived Granules in Combination with Platelet-Rich Fibrin (“Sticky Tooth”) in Socket Preservation: A Histological Evaluation

**DOI:** 10.3390/dj10020029

**Published:** 2022-02-16

**Authors:** Andreas van Orten, Werner Goetz, Hakan Bilhan

**Affiliations:** 1 Private Dental Practice Do24, Dortmunderstr. 24–28, 45731 Waltrop, Germany; andreas.van.orten@icloud.com; 2 Policlinic of Orthodontics, Centre for Dental Care, Basic Science Research in Oral Biology, Friedrich-Wilhelms University, Welschnonnenstr. 17, 53111 Bonn, Germany; wgoetz@uni-bonn.de; 3 Department of Periodontology, School for Health Sciences, Witten/Herdecke UniversityAlfred-Herrhausen-Str. 45, 58448 Witten, Germany

**Keywords:** dentin graft, autologous, PRF, socket preservation, sticky tooth, alveolar ridge preservation, bone substitute materials

## Abstract

Background: The maintenance of ridge volume following tooth extraction has gained more importance in the last few years. This clinical study aimed to assess the impact of autologous dentin particles mixed with injectable platelet-rich fibrin (i-PRF) on a sticky tooth mixture for socket preservation in terms of consecutive need for horizontal guided bone regeneration and histological findings. Methods: Eight extraction sockets in seven patients were included in this study. Autologous dentin particles were mixed with PRF, filled in the sockets, and covered with a cross-linked collagen membrane exposed to the oral cavity and fixated by crisscross sutures. An orthopantomogram was taken before the first surgical procedure and a CBCT prior to static computer-aided implant surgery. At the time of implant placement, cores were harvested with the aid of a trephine for histological examinations for every preserved socket. Results: No further horizontal GBR intervention was required in any cases, and the histological findings were unremarkable. The new bone was mostly cancellous and in direct contact with the remaining dentin granules. Conclusions: Within the limits of this clinical study, it may be concluded that this method is valuable for socket preservation and obtaining vital and good quality bone structure. The sticky tooth technique seems to be very efficient despite the more complex equipment.

## 1. Introduction

Studies on horizontal and vertical dimensional changes after tooth extraction are described in detail in the literature [1,2,3,4]. It is reported that a 25% loss of bone height and atrophy of up to 50% bucco-lingually is to be expected in the first year following extraction [1,2]. Due to the shrinkage of the ridge dimensions, a major implant placement problem may be the consequence [5].

To allow for functional and esthetic implant restorations, socket and ridge preservation techniques aim to prevent the alveolar ridge from atrophy. The basic strategy of alveolar management after tooth extractions is to preserve the volume of the alveolar socket to prevent major resorption [6,7,8]. Various techniques to maintain the volume of the alveoli after tooth extractions as far as possible have been published. Grafting materials can act through three different mechanisms that promote healing of the post-extraction socket: osteogenesis, osteoinduction, and osteoconduction [6].

In addition to surgical protocols, numerous publications describe the wide range of options for using bone replacement materials in combination with alveolar socket-preserving measures following tooth extractions. A distinction is made between autogenous, allogenic, xenogenic, phycogenic, and alloplastic augmentation materials [9].

Furthermore, to enable and support the decision-making process regarding the desired therapeutic approach, a distinction can be made between the parameters of healing time (few weeks to months), covering/closing technique (none, soft tissue graft, membranes, etc.), and the condition of the alveoli after tooth removal (intact or compromised). Often, in addition to improving the hard tissue situation, the aim is to optimize the soft tissue situation, too. Some authors describe the mere closure of the alveolar socket in terms of GBR (guided bone regeneration) without any other biomaterials or grafts.

Autologous dentin was shown as a promising material for use in socket and alveolar ridge preservation techniques in several clinical studies [10,11,12]. In fact, dentin as a graft material had been evaluated since the 1960s in terms of bone induction, although no continuous research on this topic had been conducted [13]. Dentin has great potential as a bone substitute, as it has a higher mineral content than other bone derivates. Dentin is very similar to bone in its composition: 70 percent inorganic components, 20 percent organic components, and the remaining 10 percent consists mainly of water. Hydroxyapatite and collagen are especially important structural elements. Dentin also contains several growth factors that play significant roles in bone formation, such as bone morphogenetic protein-2 (BMP-2) and transforming growth factor ß (TGF-ß). Additionally, dentin can be compared with autogenous bone in two aspects. It is osseocompatible as well as osteoinductive. These properties make dentin a valuable option as a bioactive graft material and bone substitute [14,15].

There has been an increase in evidence from human biopsies since the last decade [15,16,17]. Application of demineralized dentin in ridge preservation showed comparable osteogenesis as observed when using xenogenic bone grafting material [18]. Similarly, in a recent case report, dentin and cement granules were found to form new bone around dentin particles histologically [19]. Using ground teeth, especially autogenous dentin of patients as an augmentation material to counteract a change in alveolar volume and regenerate bone, may become an established procedure. Basic information on this was provided in several studies, while animal studies were published as early as in the 1990s [18,20,21,22]. Nevertheless, case series outside university settings and in combination with blood concentrate have been rare.

Many clinicians prefer biological additives to enhance wound healing and try to benefit from mechanisms regulating inflammation and angiogenesis [23]. Platelet-rich fibrin (PRF), which is simple in preparation and a delegable task, is one of the options [24,25]. The fibrin network in the used PRF shows many similarities with the one formed during natural healing. The content of high amounts of transforming growth factor Beta-1 (TGF beta-1), platelet derived growth factor AB (PDGF-AB), vascular endothelial growth factor (VEGF), and thrombospondin-1 from the PRF is important in stimulating some functions, such as chemotaxis, angiogenesis, proliferation, and differentiation [26,27]. Nevertheless, the efficacy of platelet concentrates in promoting wound healing and tissue regeneration is at the center of a recent academic debate.

The liquid fibrinogen has been shown to bind particulate bone grafts, which are then called “sticky bone”. This binding improves the stabilization of the particles in the defect. It adds a potential biological effect, which could accelerate the soft tissue healing process [28] and optimize the handling properties of the granules.

The purpose of this case series was to assess the capacity and the clinical feasibility of the dentin graft processed with i-PRF to an adherent, tooth-derived conglomerate for socket preservation, to assess the histological follow-ups in undisturbed wound healing, and to evaluate bone quality histologically in the healed socket. Colloquially and comparable to the term “sticky bone”, the term “sticky tooth” seems to have become established among clinicians.

## 2. Materials and Methods

Seven subjects (mean age 55.6 years; 5 male/2 female genders) with eight extraction sockets undergoing socket preservation procedures with tooth-derived granules as grafting material in a private dental practice were included in this case series (Table 1). 

The patients met the following inclusion criteria: older than 18 years, no medical history that contraindicates the surgical procedure, and at least one tooth that had to be extracted. 

The exclusion criteria were systemic diseases that might impair bone metabolism, antiresorptive therapy (as bisphosphonates), pregnancy and nursing period, psychiatric conditions, and oncologically relevant diseases. Smokers and patients with diabetes mellitus were not excluded—3 patients were cigarette smokers with daily consumption of between 10 and 20 cigarettes, whereas there were no hyperglycemic patients in the study group. 

Each patient agreed to participate in the study, providing written informed consent. No additional exposure, appointment, or other intervention was conducted for this case series. The scope of treatment followed the standard protocol of this practice. The Ethics Committee of the University of Bonn had approved the study protocol (ethical committee decision #222/05). 

All interventions were performed by the same practitioner.

The teeth had various indications for extraction (Table 1), but in all sockets the same alveolar ridge preservation protocol was applied. Multirooted teeth were separated by utilizing a small rotating Lindemann bur (H162AZ, Komet, Gebr. Brasseler GmbH; Lemgo, Germany), and the root fragments were elevated and removed with the help of matching periotomes (PT Periotomes, Hu Friedy, Chicago, IL, USA) as gently and atraumatic as possible, under local anesthesia (Ultracain DS forte, Sanofi, Paris, France). Calculus, carious lesions, remnants of the periodontal ligaments and enamel as well as restorations and any other artificial materials were removed with fast rotating carbide burs (H35L.314.012, Komet, Gebr. Brasseler GmbH; Lemgo, Germany) and curettes (Langer 3-4, American Eagle XP, American Eagle Instruments Inc., Missoula, MT, USA). The extraction socket was inspected and classified as proposed by Elian et al. in 2007 (Table 1) [29].

All surfaces of the extracted teeth and the alveoli were inspected carefully by utilizing a 5.7× magnification loupe. The remaining granulation tissue was removed by utilizing degranulation burs (EthOss, Ethoss Regeneration Ltd., Silsden, UK). The bony edges of the alveoli were uncovered utilizing a periosteal elevator. Subsequently, the patient was asked to bite on a sterile swab during the time needed to prepare the biomaterials.

The production of the particulated dentin granules was done according to the protocol described by Binderman et al. in 2014 [21]. After being dried by sterile swabs and an air syringe, the cleaned tooth particles were put into a sterile grinding chamber. The chamber was set on top of the “Smart Dentin Grinder” (KometaBio, Fort Lee, NJ, USA). A rotating rigid blade ground tooth particles inside the chamber for three seconds. The chamber’s subsequent vibration movements for 20 s led to particles between 300 µm–1200 µm by utilizing two contained sieve devices and different compartments for segregation. The process was repeated if relevant tooth particles were still left in the grinding chamber. Henceforth, the particles had been immersed in a provided cleansing agent consisting mainly of 30% alcohol and 0.5 M NaOH for ten minutes, followed by drying the particles and washing the particles with a provided sterile phosphate-buffered saline solution for three minutes (KometaBio, Fort Lee, NJ, USA). 

**Figure 1:**
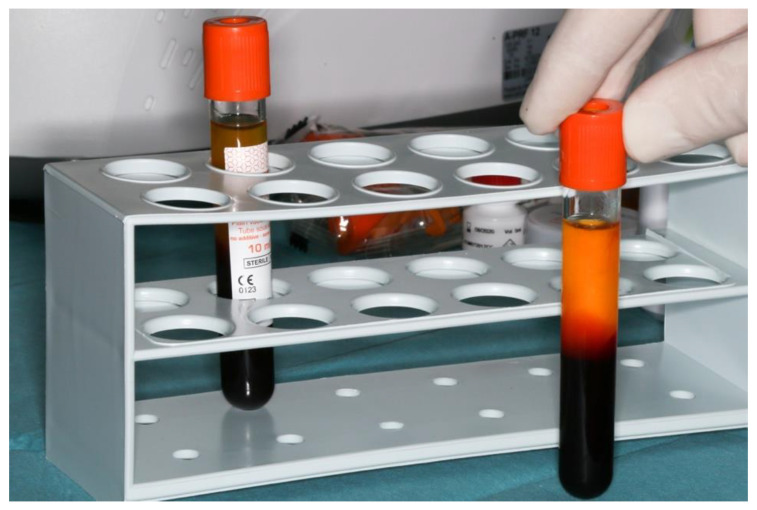
Vacuette with i-PRF preparation.

In the meantime, a temporary liquid PRF preparation following the i-PRF protocol was carried out [30]. Two i-PRF-vacutainers (Process for PRF, Nice, France) of the patient’s venous blood were collected by utilizing a 21 g needle with a butterfly handle and a vacutainer matching pump body (BC12 retractable blood sampler, Process for PRF, Nice, France). Prompt handling and speedy processing was assured (700 rpm, 3 min centrifugation time for women, 4 min centrifugation time for men because of a different hematocrit) by a dental assistant utilizing a provided centrifuge (DuoQuattro, Process for PRF, Nice, France) (Figure 1). 

**Figure 2:**
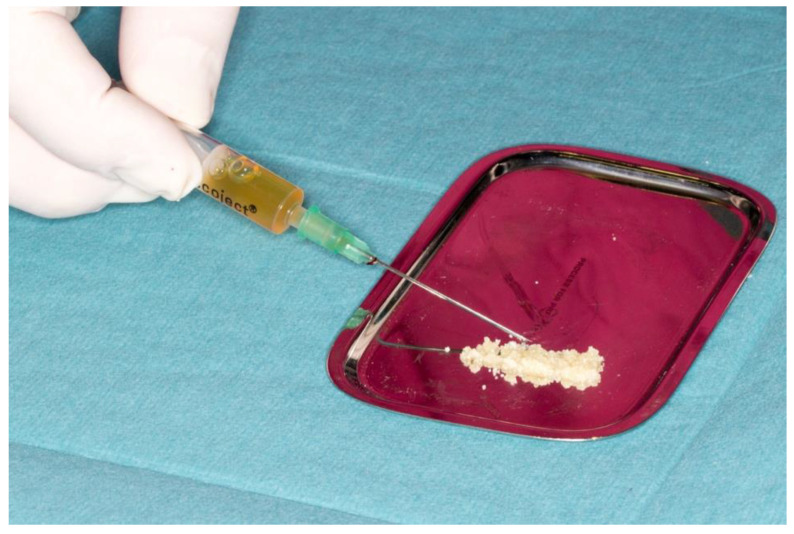
“Sticky tooth” (ground dentin mixed with i-PRF).

The liquid PRF fraction was harvested with a blunt fill needle 18 G (Becton Dickinson, Franklin Lakes, NJ, USA) and gently mixed with the dentin granules (Figure 2).

**Figure 3:**
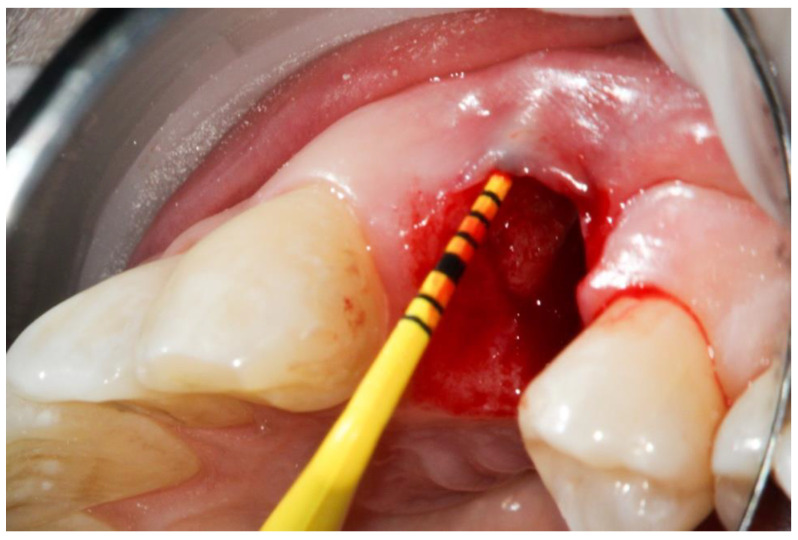
Compromised buccal lamella of tooth 14.

A native bovine collagen membrane (RCM, BioHorizons, Birmingham, AL, USA) was adapted to the alveoli carefully after hydration with the PRF liquid. Care was taken that the collagen membranes overlapped the edges of the prepared alveoli by 2 mm (Figure 3). 

**Figure 4:**
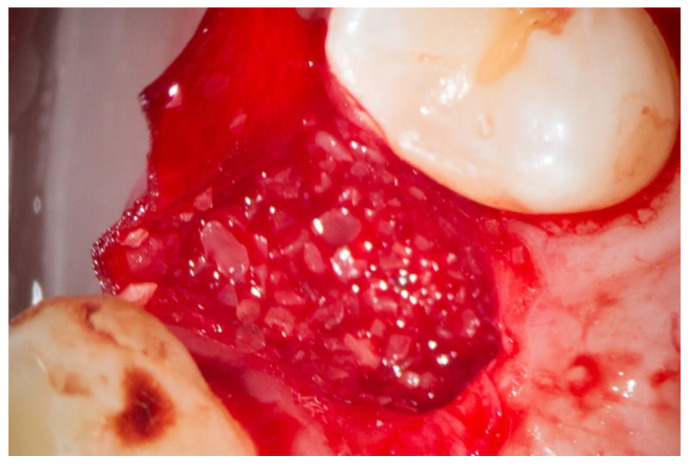
Collagen membrane and “sticky tooth” in situ.

Afterwards, the vestibular part of the membrane had been pulled back again to provide direct access to the alveoli (Figure 4). 

**Figure 5:**
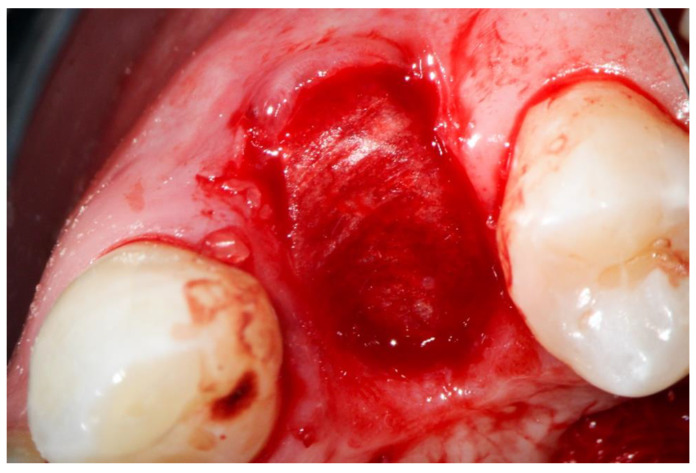
Collagen membrane adapted occlusally and palatally.

The adhering dentin granules were squeezed out and compacted with the help of a so-called PRF pad, delivered with a PRF double spoon into the alveoli, and hereinafter, again compacted utilizing a PRF compactor (Process for PRF, Nice, France). Overfilling was avoided and the membrane was put back over the osseous edges of the alveoli. In all cases, the alveoli were covered with the Mem-Lok RCM membrane (BioHorizons, Birmingham, AL, USA)—a bovine, cross-linked membrane consisting of mainly collagen type 1 fibers derived from the Achilles tendon (Figure 5).

The membranes were exposed openly to the oral cavity. A crisscross suture with a 6-0 resorbable thread (Monofast, Mectron, Carasco, Italy) provided temporary stability for the membrane and a slight adaptation of the wound margins.

The patient was provided with NSAR for analgesia (600 mg Ibuprofen, Ibuflam, Zentiva, Pharma GmbH, Berlin, Germany). The post-op regimen included the patient’s instruction to abstain from mechanical plaque control in the treated area for one week and use chlorhexidine (Chlorhexamed GlaxoSmithKline Consumer Healthcare GmbH & Co. KG, Munich, Germany) mouth rinse (0.2%) twice a day instead.

**Figure 6:**
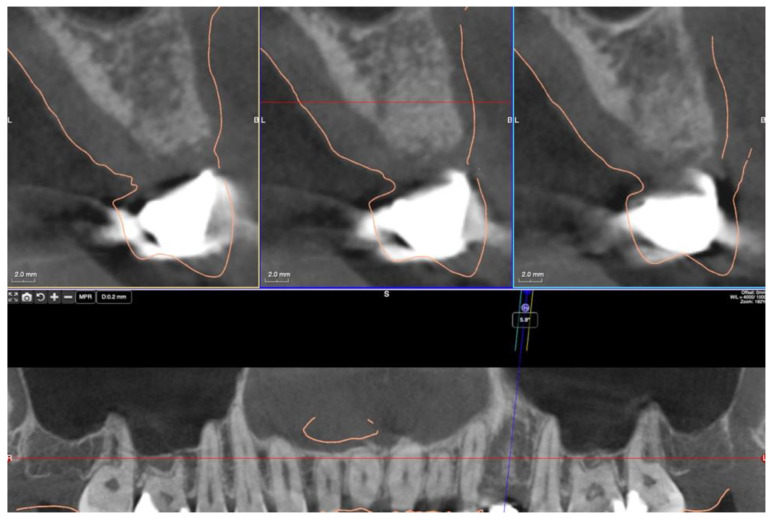
CBCT view six months post-augmentation before implantation in region 24.

A healing time of at least 15 weeks was intended to provide a solid implant site. A presurgical assessment of the alveolar ridge was conducted by CBCT (Orthophos XG 3D, Dentsply Sirona, York, PA, USA) also to facilitate flapless surgery by fabricating a static computer-aided implant surgery template (Figure 6).

After applying a local anesthetic (Ultracain DS forte, Sanofi, Paris, France) the implant site was prepared with a punch when ≥8 mm of keratinized tissue had been provided. The site was then prepared with a mid-crestal incision to ensure a sufficient level of keratinized tissues afterwards. The implant site was prepared with a trephine bur (Trephine Ejection Kit, Hager & Meisinger GmbH, Neuss, Germany) and the intended implant placed. After carefully removing the bone core from the trephine, it was stored in a buffered 10% formalin solution.

### Histological Analysis

Biopsies collected from patients were processed according to standardized histological protocols. After formalin fixation and decalcification in EDTA and paraffin embedding, serial sections were made and selected for further processing with hematoxylin-eosin (HE). Subsequently, the specimens were examined under the microscope, generally in ×20 magnification.

## 3. Results

### 3.1. Bone Quality

**Figure 7:**
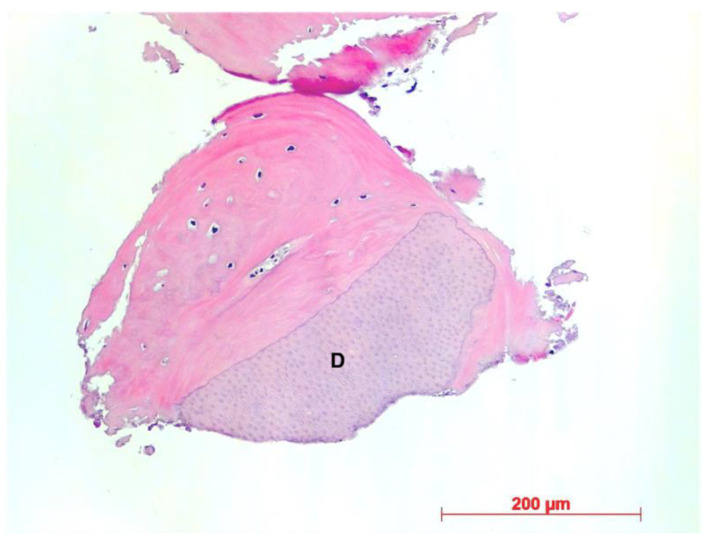
Patient 1—Dentin granule (D) embedded into newly formed bone, HE, original magnification ×20.

**Figure 8:**
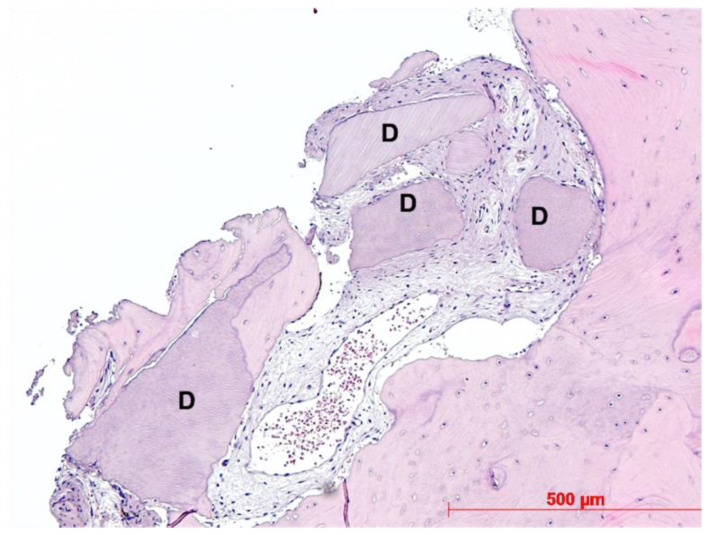
Patient 2—Newly formed bone, dentin granules (D) with and without osteogenesis, HE, original magnification ×20.

**Figure 9:**
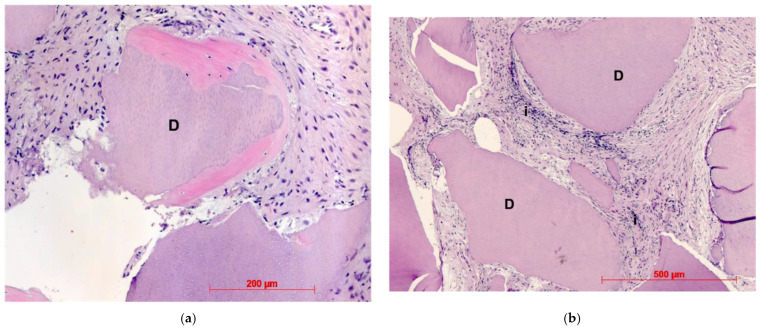
(**a**): Patient 3—Early-stage osteogenesis around dentin granule (D), HE, original magnification ×20. (**b**): Patient 3—Dentin granules (D) without osteogenesis, i = infiltration, HE, original magnification ×20.

**Figure 10:**
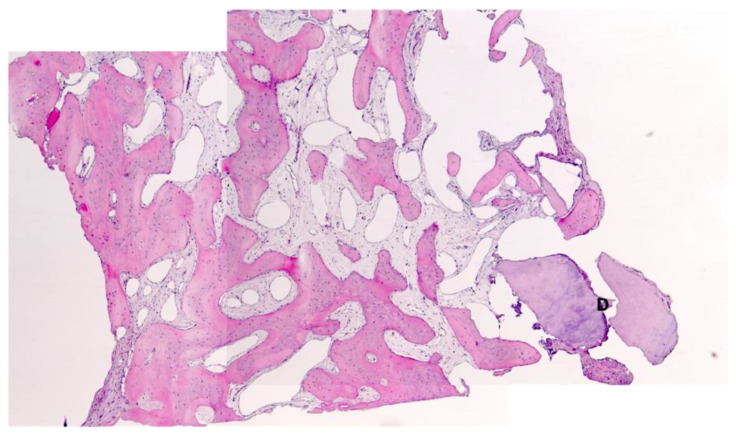
Patient 4—Newly formed cancellous bone, D = dentin granules, HE, original magnification ×5.

**Figure 11:**
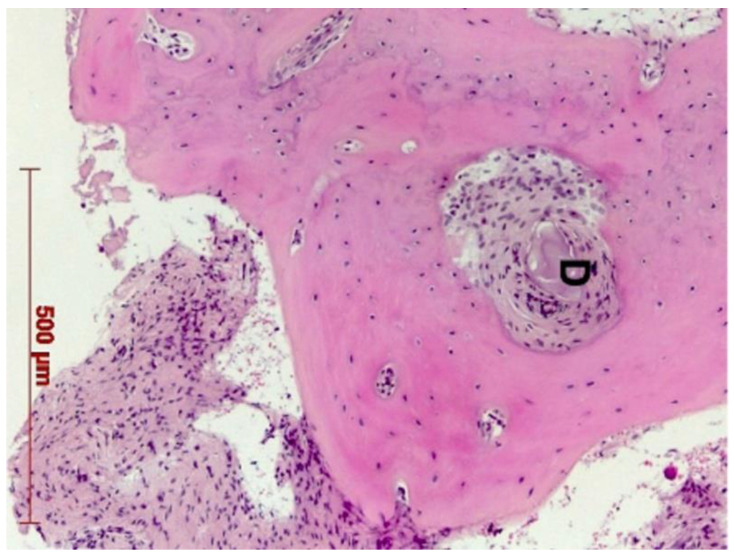
Patient 5—Early-stage osteogenesis around dentin granule (D), HE, original magnification ×20.

**Figure 12:**
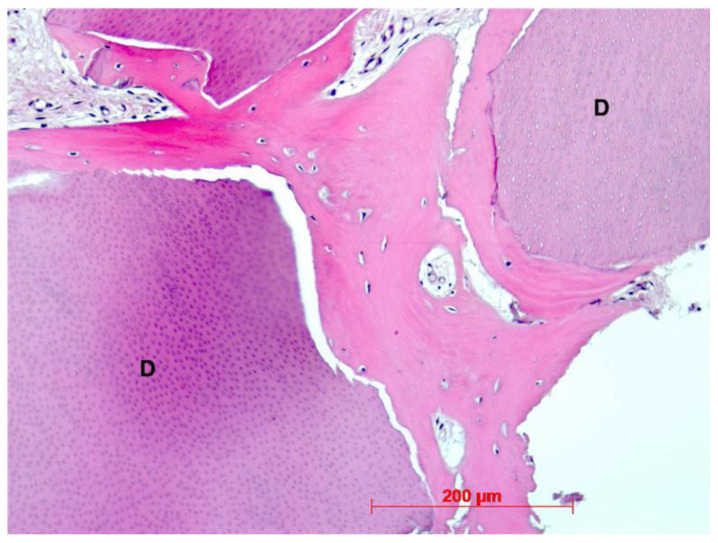
Patient 6—Perigranular osteogenesis between dentin granules (D), HE, original magnification ×20.

**Figure 13:**
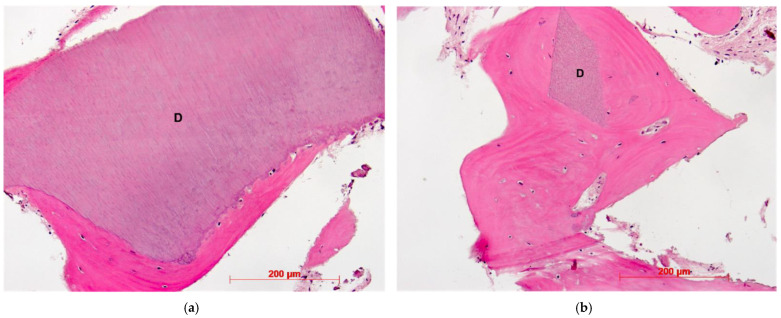
(**a**): Patient 7—Early-stage osteogenesis around dentin granule (D), HE, original magnification ×20. (**b**): Patient 7—Dentin granule (D) remnant embedded into newly formed bone, HE, original magnification ×20.

Histological evaluation showed bone formation, which was mostly present in intimate contact with the surface of the dentin particles and surrounding them. Moreover, all specimens presented mostly cancellous bone and osteogenic activity. Dentin graft particles were still visible and had not been resorbed by osteoclastic activity. (Figure 7, Figure 8, Figure 9, Figure 10, Figure 11, Figure 12 and Figure 13).

### 3.2. Ridge Dimension

**Figure 14:**
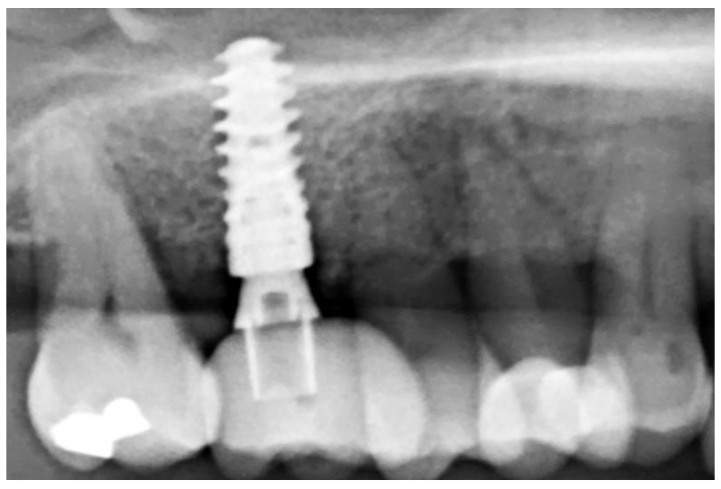
Radiological follow-up after 26 months.

During the subsequent insertion of dental implants, no additional bone augmentation was necessary—except for space making during sinus floor elevation procedures (Figure 14), pointing to the reached clinical target of “preserving ridge dimension for later implant placement”.

## 4. Discussion

At the time point of tooth extractions, two different options are available for implant placement to avoid dimensional ridge alterations hereafter: immediate implant placement with or without bone regeneration or delayed implantation combined with socket preservation. Socket preservation may prevent 1.5–2.4 mm of horizontal and 1–2.5 mm of vertical mid-buccal bone resorption [31]. At present, there is no evidence of the superiority of a given biomaterial over the others in terms of socket preservation. Xenogenic bone substitute materials seem to be the first choice for the volumetric preservation after tooth extraction. Still, they seem to be inconsistent concerning the question of new bone volume gain, especially bovine bone substitute materials which seem to be inferior. In contrast, porcine bone substitute materials were related to higher new bone gains. Allograft materials were not superior to the control group of unassisted healing, while CaS and ß-TCP resorbed faster than other biomaterials [32]. The prevalence of vegetarianism varies worldwide from 4–8% in the US, Canada, and Germany up to 30% of the people in India, mostly for ethical concerns about animal welfare [33]. The acceptability of different xenogenic substitute materials due to religious beliefs can also lead to conflicts between patients and surgeons [34]. Autologous alternatives may be able to defuse this conflict.

The focus of these case series with consecutive histological examinations is the desire to contribute in small part to the discussion on a useful combination of autologous preparations for socket preservation after tooth extractions under dental practice conditions. The opportunity of providing an autologous bone substitute material without donor site morbidity may lead to an improved patient acceptance for alveolar ridge and GBR procedures. In addition to existing methods and bone replacement materials (autogenous, allogenic, xenogenic, phycogenic, and alloplastic) with their different potentials regarding osteogenic, osteoinductive, and osteoconductive properties, another treatment option is described that may allow for sufficient bone regeneration of the alveolar ridge with safe, predictable, and easy-to-learn protocols.

Most of the studies with promising results using ground dentin graft as bone graft substitute, pointed out the need for studies identifying the bone remodeling properties of dentin through histological and histomorphometric analysis. This need was one of the driving factors for us to undertake this investigation, although the contribution of a small sample size is limiting.

Our findings show sufficient bone regeneration histologically in all cases. Furthermore, true osteogenesis with formation of woven or fibrous bone, which has already been partially transformed into mature lamellar bone without signs of inflammation or necrosis could be verified. In the majority of cases, osteoblasts have also been detected as signs of active bone.

In all eight specimens dentin particles were observed on which perigranular osteogenesis took place, as already described in a recent study [19]. Based on these findings, we can at least assume an osteoconductive effect of the mineralized dentin fragments. An osteoinductive effect may also be assumed—which could be verified by further investigations of the specimen by proving the presence of osteopontin, osteocalcin, and runx2—but this was not the object of this investigation. However, a complete osteoclastic resorption of the particles was not observed in this stage. Further histological examinations, after the dentin fragments had been left in place for a longer period, would have to be carried out to allow for statements about their possible degradation.

The capacity of dentin in bone formation may be explained by the same embryologic origin: the organic as well as the inorganic composition of dentin is very similar to bone [35]. Dentin has been shown to be involved in the bone remodeling process with its osteoconductive and osteoinductive properties [15,36,37]. Since the organic matrix dominated by Type I collagen fibers also presents non-collagen proteins such as phosphoproteins, osteocalcin, proteoglycans and glycoproteins, several studies have been conducted to test dentin as a potential biomaterial and bone substitute. It was shown that calcium and phosphates are still present within the collagen components even after the particle cleaning procedures that are conducted before use [38]. It was reported in several studies that the dentin graft was replaced more homogeneously by newly formed bone than in the autogenous bone grafts, concluding that the roots of extracted teeth revealed a structural and biological potential to work as an autograft alternative to autogenous bone [39,40], and even supported the early stages of osseointegration after implant placement very similar to autologous bone [41]. As pointed out in a recent systematic review, studies evaluating autologous teeth as grafting material involved only small patient numbers and short follow-up periods. Nevertheless, results are promising, and the dentin graft may be a valuable alternative biomaterial [42]. In a recent study, histologic samples from grafted areas at varying intervals were examined, to allow observation of bone-healing dynamics over time. These samples were occupied by dentin particles that had begun to connect via bridges of woven bone at 3 months post healing, and vital bone was in direct contact with residual particles with no inflammatory infiltrate [43].

In a recent study extracted teeth alone and extracted teeth mixed with equal quantity of xenograft were compared; the histologic analysis revealed no inflammatory or infective reaction against both groups, whereas the histomorphometry results between the two groups were different. The group with the dentin graft showed an amount of new bone greater than the other group (+85.29%) and a minor quantity of residual graft (−83.59%), thus concluding that dentin alone shows a larger amount of new bone [44].

The literature shows predominantly positive characteristics in the use of blood concentrates, such as PRF preparations, in both bone and soft tissue regeneration [23]. Recent histomorphometric studies have demonstrated higher bone formation in the test group using PRF preparations for mandibular preservation compared to the control group [12,45]. It was reported in detail on different indications of PRF processing in connection with preventive and augmentative measures in dentistry [30]. Depending on the patient-specific defect morphologies and defect sizes, blood concentrates such as PRF preparations (A-PRF, i-PRF, and several other process protocols) can be used alone or in combination with bone replacement materials. Various clinical studies have already confirmed the reliability of alveolar management and sinus lift with blood concentrates and PRF concentrates [30]. It was shown that PRF can release a variety of growth factors (VEGF, EGF, TGF-β, PDGF) in high concentrations, thereby supporting cascades such as angiogenesis or epithelialization and positively influencing the recruitment of further cells. This appears to have advantages in both hard and soft tissue healing although PRF alone is not capable of preventing alveolar ridge dimensional changes post extractions over time [46]. The necessity that an acquisition of a centrifuge is an essential prerequisite to produce PRF-related biomaterials should not remain unmentioned. The effort of the process sequence and the related costs can be seen as a minor obstacle: the drawing and processing of blood is a delegable step which can be provided by a dental assistant.

In a very recent study, where socket preservation post flapless tooth extraction with mineralized dentin particulate autograft and chopped platelet-rich fibrin was evaluated by CBCT comparisons of before and after, it was concluded that this technique and biomaterial combination had resulted in well-maintained vertical socket dimensions and minimal horizontal ridge reduction [47]. It has been concluded that autogenous dentin graft has bone formation capacity on early period of bone healing in a very recent clinical study where extraction sockets were randomly treated either with autologous dentin with PRF, only autologous dentin or left just with the blood clot for natural healing [48]. It was stated that it can be used as bone graft material in augmentation procedures and its combined use with PRF accelerates new bone formation. Recent studies had shown that liquid PRF promoted greater regeneration potential of cells [49,50], whereby Wang et al. [50] had reported that it stimulates greater cell migration, proliferation, and collagen synthesis when compared to platelet-rich plasma. Nonetheless, the biological activation of bone grafts using injectable platelet-rich fibrin was described in a recent article [51] and supporting the results presented here. Clinicians who prefer a faster polymerization of the i-PRF and who want to achieve a stronger sticky tooth or bone preparation tend to add fibrinogen to the liquid PRF which can be collected during the preparation of solid PRF membranes and following the principles of the S-PRF protocol.

There is a grey area about the parts of the teeth to be ground and not much evidence-based information on whether only dentin or the whole tooth give better results as a graft. In a very recently published article, the histological and histomorphometrical results between vital whole and non-vital endodontically treated teeth used as autologous grafts in post-extractive socket preservation procedures, revealed no statistical differences comparing whole and endodontically treated teeth in bone regeneration [52]. Similar to the necessity of acquiring a centrifuge for the utilization of PRF-related biomaterials, the need of providing the hardware in order to grind teeth should be mentioned. Similar to PRF preparations the further effort of processing teeth for subsequent use as a bone substitute material is rather low. The process is completely delegable, and the costs of the disposable items are rather low.

In the case series shown here, an attempt was made to generate an advantage for the patient in terms of quality of bone regeneration and volume preservation when using both approaches—the use of PRF preparations and the use of autogenous dentin particles. There is an advantage for the dentist when using PRF preparations because mixing autogenous dentin with PRF and fibronectin produces a malleable conglomerate, the so-called “sticky tooth” after a short time, which has the benefit that dislocation of particles is generally no longer observed.

In this case, series attention was given not to force a primary closure to cover the grafts with flap elevation to avoid the loss of vestibular sulcus depth especially in the vestibular part, which could complicate future interventions [53].

The possibility of providing an autologous option, which is highly osteogenic and does not have the disadvantage of an autologous bone which may be too quickly resorbed, might be an additional alternative for patients with an aversion to allogenic or xenogenic graft materials. Another advantage in comparison to autologous bone harvesting procedures is that there is no donor site comorbidity, which may reduce post-operative complaints and complications drastically. The clinician is not dependent on prefabricated biomaterials from manufacturers and may reduce material costs.

Future research with volumetric comparisons of the ridge dimensions before tooth extraction and after healing with socket preservation will shed light on the success of a particular grafting material and a special technique. The histologic and histomorphometric analysis gives without a doubt valuable information concerning the bone quality of the socket ossification. To show the success of the particulate dentin graft in comparison to other biomaterials, randomized clinical trials with split-mouth design will be valuable.

## 5. Conclusions

Within the limitations of this case series with histological evidence it may be concluded that the combination of ground autogenous dentin combined with PRF preparations could represent a good alternative for socket preservation procedures to maintain the volume of the former socket with a high regenerative potential. This may lead to subsequent simplified surgical approaches and a higher patient acceptance for dental implant procedures. Long-term observations with a higher number of cases, as well as the evaluation of the demineralized dentin graft procedure would be recommended to draw more reliable conclusions.

## Figures and Tables

**Table 1:** Descriptive data about the “socket preservation” cases.

Patient	Gender	P. Age	Reason for Tooth Loss	Time to Biopsy/d	Implant Site	Socket Type
#1	f	28	Root fracture/endodontic complication	211	24	3
#2	f	50	Periodontal complication	420	16	3
#3	m	57	Periodontal complication	106	12	3
#3	m	57	Periodontal complication	106	22	3
#4	m	73	Periodontal complication	134	25	3
#5	m	46	Root fracture/endodontic complication	195	26	2
#6	m	69	Combined endo-perio complication	158	17	3
#7	m	66	Root fracture/endodontic complication	196	35	1

## Data Availability

Data available on request due to restrictions privacy. The data presented in this study are available on request from the corresponding author. The data are not publicly available due to data protection.
